# Editorial: Methods in genome, pan-genome, pan-transcriptome, and gene regulatory network (GRN) construction and analysis

**DOI:** 10.3389/fpls.2023.1152708

**Published:** 2023-02-24

**Authors:** Parul Gupta, Song Li

**Affiliations:** ^1^ Department of Botany and Plant Pathology, Oregon State University, Corvallis, OR, United States; ^2^ School of Plant and Environmental Science, Virginia Tech, Blacksburg, VA, United States

**Keywords:** pangenome, pan-transcriptome, gene regulatory network (GRN), GWAS - genome-wide association study, weighted gene co-expression analysis (WGCNA), SNP, RNA-seq, crop improvement

In the past two decades, the cost of genomic sequencing has been declining and many reference genomes have been sequenced for economically important plant species. With this rich resource of genomic sequences, and the improving accuracy of long read sequencing technologies, pan-genome assembly and analysis has become more feasible and new resources with novel insight from re-sequenced genomes are emerging. The pan-genome represents the entire set of genes within a species, including a core genome and the dispensable genome. Pan-genome assembly and analysis are helpful in analyzing various diverse traits within species. For example, pan-genome and comparative genomic analysis revealed that two different mechanisms existed for domestication and de-domestication processes of rice and its wild relatives (Ma et al.).

The goal of this Research Topic was to present the community with new methods and analytical solutions for breeding, crop improvement and domestication. We are honored to receive submissions of five manuscripts which are in line with the aim of this Research Topic and cover a wide range of research interests. These published manuscripts include research about de-domestication of weedy rice (Ma et al.), genetic resources for breeding antioxidant-rich cultivars for grape species (Park et al.), molecular mechanism for accumulation of organic acids in Chinese dwarf cherry (Guo et al.), genetic architecture of fruit branch angle in cotton (Shao et al.), and gene regulatory network involved in anthocyanins biosynthesis in maize pericarp (Li et al.).

Genome resequencing of hundreds of varieties or accessions of a single species can help in identification of single nucleotide polymorphism (SNPs) and structural variations (Huang and Han, 2014). With a SNP map, the phenotypic variations can be correlated with genetic variations using genome wide association studies (GWAS). GWAS is useful for identification of genetic variants associated with specific trait. High-throughput sequencing technologies (whole genome resequencing, RNA-Seq, single-cell sequencing) revolutionized the power and resolution of GWAS. Post-GWAS analyses include various new approaches for deriving more meaningful information, however, prioritization of candidate gene/s for complex traits is very challenging. In this Research Topic, GWAS was performed for Muscadine grape (Park et al.) and cotton (Shao et al.). Candidate genes related to chemical composition (grape) and shoot architecture (cotton) were identified. In addition to genetic analysis, in both studies, candidate genes were identified by combining GWAS results with transcriptome data of selected varieties with contrasting phenotypes. In cases where the whole genome association studies were not possible, transcriptome analysis using RNA-seq can be performed using selected varieties at specific tissue type of interest. Differentially expressed genes as well as co-expression network analysis have been used in Chinese cherry fruit (Guo et al.) and maize pericarps (Li et al.). Candidate genes were selected using differentially expressed genes as well as co-expression modules in both the studies.

Muscadine grape is native to the southeastern United States, and this type of grape is gaining popularity due to its nutritional and health values. To facilitate breeding of Muscadine grapes with enhanced nutrient profile, Park et al. applied a multi-locus GWAS study of 350 muscadine genotypes to identify genetic markers that are associated with multiple antioxidant-related traits including total phenolic content, total flavonoid content, and free radical scavenging activity in muscadine berry skin. Through the GWAS analysis, 12 Quantitative Trait Nucleotides (QTNs) were identified from the four traits. Combined with transcriptome analysis, two candidate genes positively and negatively correlated to the quantitative property of antioxidant activity were found to be co-localized with the genetic markers (QTNs).

Chinese dwarf cherry is a fruit enriched with nutrients such as calcium, protein, vitamins, and organic acids. The acidity of the cherry fruit is a key factor that affects consumer acceptance. Guo et al. studied the molecular basis for the differences in organic acid contents in Chinese dwarf cherry fruits using comparative transcriptomics and weighted gene co-expression network association analysis (WGCNA) approach (Langfelder and Horvath, 2008). Two varieties with contrasting organic acid levels were used to collect transcriptome data at different developmental stages. Differential gene expression analysis in combination with WGCNA revealed candidate genes and Gene Regulatory Network (GRN) related to the organic acids content in the fruits of these two cultivars. This research provides a foundation of understanding the mechanism of acid regulation in the Chinese dwarf cherry fruit.

Plant architecture is a key agronomic trait for many plant species. Fruit branching angle (FBA) for cotton is particularly important for the efficiency of mechanical harvesting. In cotton plants, the FBA affects plant density, photosynthetic efficiency, disease resistance, and yield of cotton fiber. To investigate the molecular basis of FBA in cotton, Shao et al. used a collection of 163 cotton varieties to perform whole genome resequencing. A combination approach of GWAS and RNA-seq methods helped in identification of 55 SNPs and 18 candidate genes associated to FBA trait. FBA phenotype was measured in 4 different environments and pathways associated with FBA and genes in response to gravity and light were identified.

Anthocyanins are the major pigment compound in maize and many other plant species. It has been shown that the types of anthocyanins in maize are similar between different varieties, but the level of different anthocyanins are the major factor that contributes to the difference in kernel color (Moreno et al., 2005). In this study, Li et al. performed a short time course transcriptome analysis of two varieties of maize with transparent and anthocyanins-enriched pericarps. WGCNA was used to identify ten modules, and one module was determined as the most relevant module, because of the correlation of this module to the levels of anthocyanins. This study discovered four structural genes and one transcription factor (*Lc* gene) involved in anthocyanins biosynthesis. Most interestingly, a *Lc* gene (*Zm00001d026147*) was predicted as a key regulator of other enzymes involved in anthocyanins metabolism. Yeast one-hybrid experiment was conducted to confirm the interaction of the regulator with its target genes.

Plant pan-genomes have become available for multiple species such as rice (Zhao et al., 2018), maize (Hufford et al., 2021), soybean (Liu et al., 2020) and tomato (Gao et al., 2019). However, many useful species have not been sequenced in these large consortia. For example, weedy rice (*Oryza sativa f. spontanea*) is an important wild rice species with useful agronomic traits such as biotic and abiotic tolerance. The genome of weedy rice can provide a useful genomic resource to the breeding community as well as functional genomic studies. Ma et al. performed whole genome sequencing of a weedy rice variety (A02) with Nanopore long read sequencing platform. More than 5,000 structural variations (SVs) were found in this variety by comparing it to the reference genome of Nipponbare. The de-domestication and domestication sites of weedy-rice were identified using a pan-genome analysis with 238 rice accessions (weedy and cultivated). This study can serve as a foundation to unravel evolutionary aspects of wild, cultivated, and weedy rice to help breeding projects.

In summary, papers in Research Topic illustrate data-driven approaches enabling phenotyping, identification of genes related to agronomically important traits, domestication of crops, and improvement of nutrients quality. Availability of such genomic resources provide an expansion of toolbox for plant breeding, and crop improvement projects. Previously, analysis of genetic architecture of agronomically important traits, for example - quantitative trait locus (QTL) mapping and GWAS, were primarily based on SNP markers. Reference quality genome assemblies and pangenomes provide deeper understanding of structural variations associated with many important agronomic traits including crop domestication and provide new heights to crop genomics.

## Author contributions

All authors listed have made a substantial, direct, and intellectual contribution to the work and approved it for publication.
